# Nesfatin-1 facilitates IL-1β production in osteoarthritis synovial fibroblasts by suppressing miR-204-5p synthesis through the AP-1 and NF-κB pathways

**DOI:** 10.18632/aging.203559

**Published:** 2021-09-24

**Authors:** Kun-Tsan Lee, Bo-Cheng Chen, Shan-Chi Liu, Yen-You Lin, Chun-Hao Tsai, Chih-Yuan Ko, Chih-Hsin Tang, Kwong-Chung Tung

**Affiliations:** 1 Department of Veterinary Medicine, College of Veterinary Medicine, National Chung-Hsing University, Taichung, Taiwan; 2 Department of Orthopedics, Taichung Veterans General Hospital, Taichung, Taiwan; 3 School of Medicine, China Medical University, Taichung, Taiwan; 4 Department of Medical Education and Research, China Medical University Beigang Hospital, Yunlin, Taiwan; 5 Department of Sports Medicine, College of Health Care, China Medical University, Taichung, Taiwan; 6 Department of Orthopedic Surgery, China Medical University Hospital, Taichung, Taiwan; 7 Chinese Medicine Research Center, China Medical University, Taichung, Taiwan; 8 Department of Medical Laboratory Science and Biotechnology, College of Medical and Health Science, Asia University, Taichung, Taiwan

**Keywords:** osteoarthritis, nesfatin-1, interleukin-1 beta, miR-204-5p, synovial fibroblasts

## Abstract

The progression of osteoarthritis (OA) is mediated by adipokines, one of which is nesfatin-1, which is responsible for the production of inflammatory cytokines. However, how this molecule may affect the synthesis of the proinflammatory cytokine interleukin 1 beta (IL-1β) in OA is unclear. Our analyses of records from the Gene Expression Omnibus (GEO) dataset and clinical specimens of synovial tissue revealed higher levels of nesfatin-1 and IL-1β in OA samples compared with normal healthy tissue. We found that nesfatin-1 facilitates IL-1β synthesis in human OA synovial fibroblasts (OASFs) and suppresses the generation of micro-RNA (miR)-204-5p, as the miR-204-5p levels in OA patients were lower than those in healthy controls. Nesfatin-1-induced stimulation of IL-1β in human OASFs occurred via the suppression of miR-204-5p synthesis by the PI3K, Akt, AP-1 and NF-κB pathways. We suggest that nesfatin-1 is worth targeting in OA treatment.

## INTRODUCTION

The main feature of osteoarthritis (OA) is the disease-related increase in inflammatory mediators in the synovial membrane leading to pannus formation and resulting in cartilage degradation and bone destruction [1, 2]. The generation of proinflammatory cytokines and chondrolytic enzymes in the inflamed synovial tissue and cartilage degradation promotes inflammation in the synovium [3–5]. OA synovial fibroblasts (OASFs) enhance arthritic pathology by increasing levels of chondrolytic enzymes and inflammatory cytokines [6, 7].

Anti-inflammatory drugs, such as corticosteroids and NSAIDs, are typically the first choice of medication to lower ongoing inflammation and ameliorate the pain associated with arthritis [8, 9]. The low-grade, chronic inflammation experienced by patients with arthritis perpetuates the release of proinflammatory mediators that cause ongoing damage in the synovium, bone and cartilage [10, 11]. Among these inflammatory mediators, interleukin 1 beta (IL-1β) contributes to the pathogenesis of OA by facilitating proteolytic enzyme-induced damage in the cartilage extracellular matrix [2]. Levels of IL-1β in OA synovial fluid and serum are higher than those in normal healthy individuals and have therefore been targeted by therapies such as the IL-1β inhibitor canakinumab [12]. Reducing the activity of proinflammatory cytokines has been shown to slow the progression of arthritis [2], as these cytokines and also matrix- and cartilage-degrading enzymes account for pathohistological changes that occur in OA [13–15]. These inflammatory mediators are regulated by different signaling pathways in OA, such as the phosphoinositide 3-kinase (PI3K), protein kinase B (Akt), activator protein 1 (AP-1), and nuclear factor kappa-light-chain-enhancer of activated B cells (NF-κB) pathways [16]. In particular, the PI3K/Akt/mTOR signaling pathway is essential for maintaining joint health, and correlates with the degradation of cartilage in OA pathogenesis, as this pathway is involved in chondrocyte apoptosis, proliferation and cytokine production [16]. The phosphorylation of PI3K/Akt signaling can promote the translocation of AP-1 and NF-κB into the nucleus and increases gene expression of proinflammatory mediators, such as prostaglandin E2 and cyclooxygenase-2 [17–19]. Thus, examining the PI3K/Akt, AP-1 and NF-κB pathways is expected to improve our understanding as to how to reduce inflammatory cytokine expression in OA.

As the progression of OA is regulated by numerous microRNAs (miRs), modulating miRNA-mediated proinflammatory cytokine expression represents one therapeutic strategy for OA [20–23]. We have previously described how several miRNAs (including miR-let-7c-5p, miR-149-5p and miR-144-5p) inhibit the progression of OA disease [24, 25]. Similarly, other research has shown that the targeting of runt-related transcription factor 2 (Runx2) by miR-204-5p downregulates chondrocyte proliferation and ameliorates the OA development in rats [26]. Moreover, miR-204-5p downregulates levels of tumor necrosis factor-α (TNF-α), IL-6 and prostaglandin E2 and thereby decreases inflammatory responses in IL-1β-treated human OASFs [27].

Adipose tissue is an endocrine organ that produces hormones as adipokines including adiponectin, leptin, resistin and nesfatin-1 [28]. Nesfatin-1 plays a crucial role in food intake and weight control [29] and regulates different cellular functions such as growth, migration, differentiation and apoptosis in mammalian cells [30]. Nesfatin-1 levels in serum and synovial fluid correlate with the severity of knee OA [31]. Nesfatin-1 also suppresses the destruction of cartilage and ameliorates OA in rats [32]. However, the effects of nesfatin-1 on IL-1β expression in OASFs is unknown. In this study, our comparison of nesfatin-1 and IL-1β levels found that both were higher in OA patients than in healthy normal controls, and our investigations revealed that nesfatin-1 promotes IL-1β synthesis in OASFs by suppressing miR-204-5p expression in the PI3K, Akt, AP-1 and NF-κB pathways. Targeting nesfatin-1 levels in synovial fibroblasts may assist in the management of OA disease.

## RESULTS

### Higher levels of nesfatin-1 and IL-1β expression in OA patients

**Figure 1:**
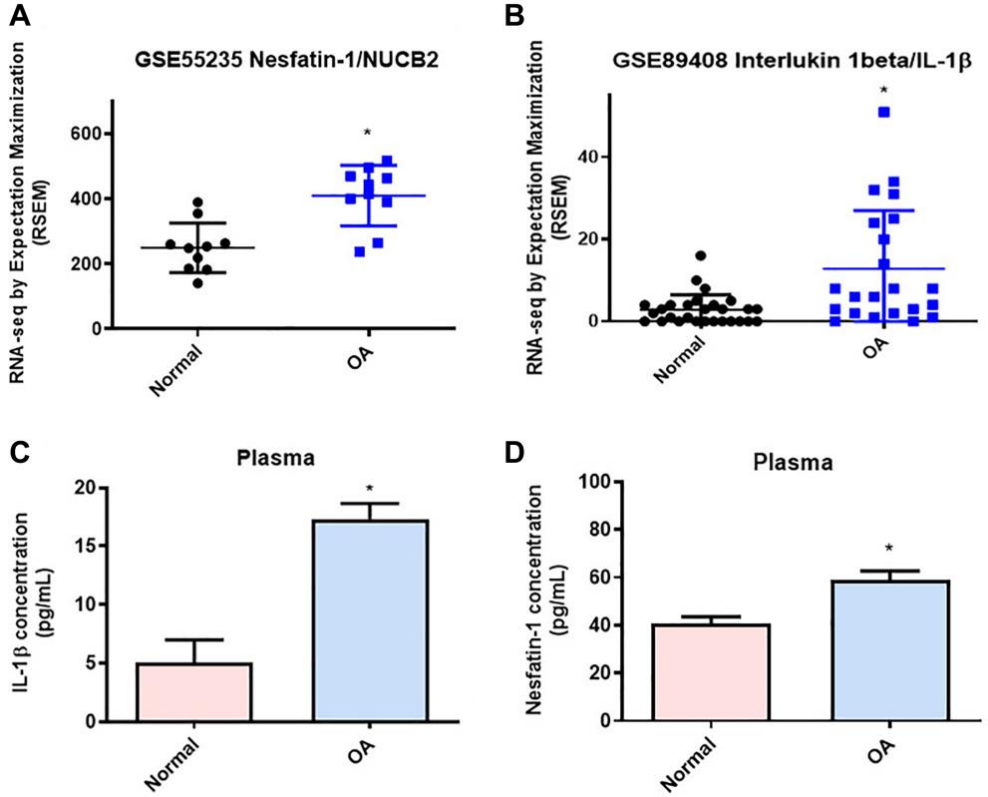
**Higher levels of nesfatin-1 and IL-1β in OA synovial tissue than in tissue from healthy controls.** (**A** and **B**) Levels of nesfatin-1 and IL-1β in normal and OA synovial tissues retrieved from the GEO dataset. (**C** and **D**) ELISA analysis showing higher serum levels of nesfatin-1 and IL-1β among OA patients compared with healthy controls. ^*^p < 0.05 compared with normal synovial tissue.

Concentrations of nesfatin-1 in human serum and synovial fluid reflect the severity of OA [31]. Gene Expression Omnibus (GEO) database records revealed higher levels of nesfatin-1 and IL-1β in synovial tissues from OA patients compared with samples from healthy controls (Figure 1A and 1B). Our clinical samples also confirmed higher levels of nesfatin-1 and IL-1β in serum from OA patients compared with serum from healthy individuals (Figure 1C and 1D), indicating that nesfatin-1 and IL-1β are associated with progression of OA.

### Nesfatin-1 increases IL-1β synthesis in human OASFs through the PI3K, Akt, AP-1 and NF-κB signaling pathways

**Figure 2:**
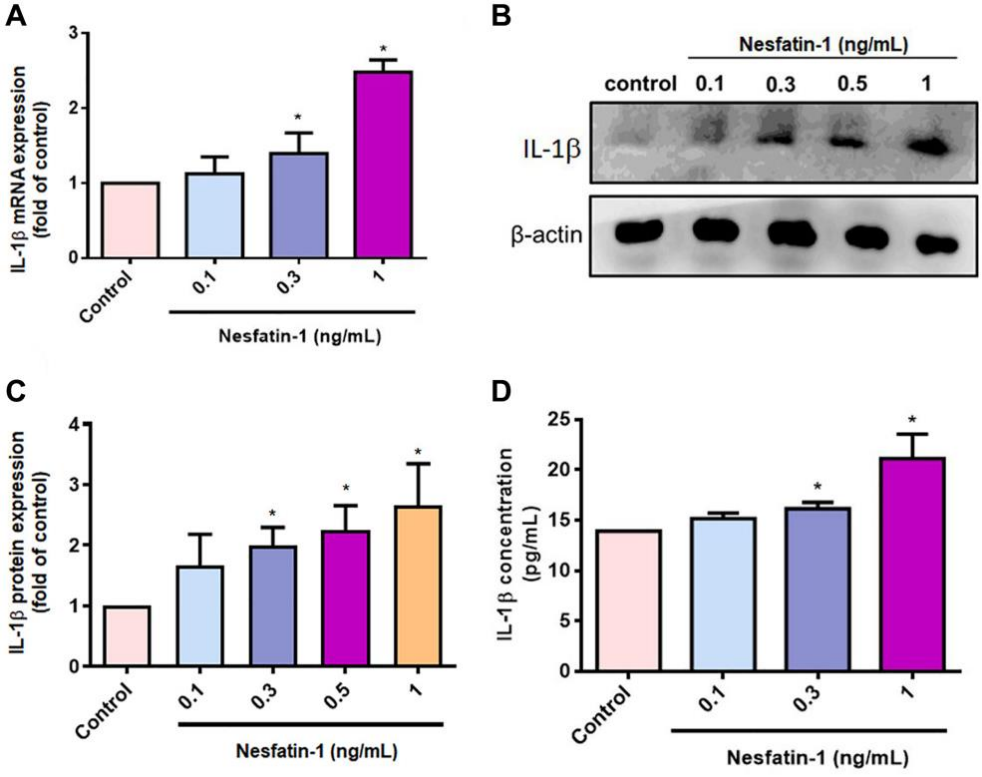
**Nesfatin-1 enhances IL-1β production in human OASFs.** OASFs were incubated with nesfatin-1 (0.1–1 ng/mL) and IL-1β mRNA and protein expression was examined by qPCR (**A**) and Western blot (**B**). Quantitative data for Western blot (**C**) and ELISA (**D**) assays. ^*^p < 0.05 compared with the control group.

**Figure 3:**
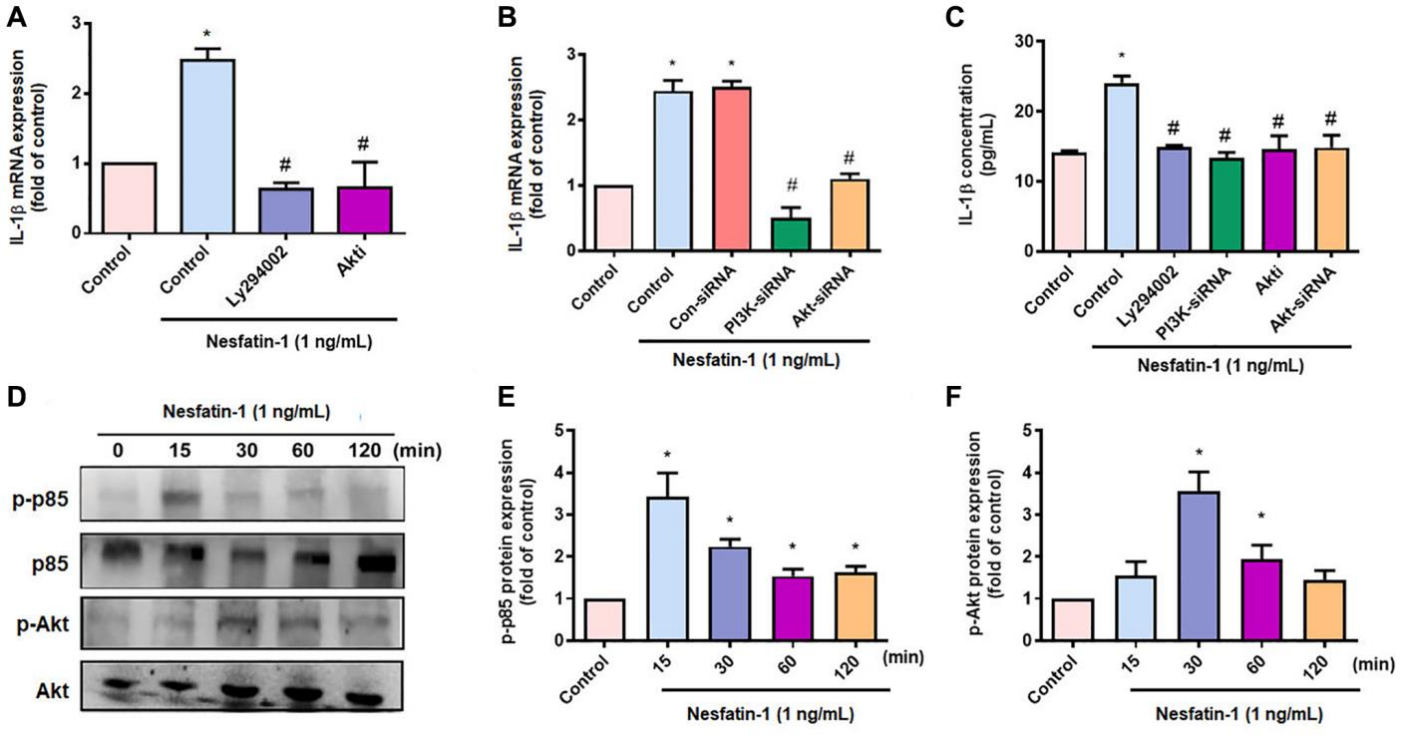
**The PI3K/Akt pathways mediate nesfatin-1-induced stimulation of IL-1β synthesis.** (**A**–**C**) OASFs were treated with a PI3K inhibitor (Ly2942002) or Akt inhibitor (Akti), or transfected with p85 or Akt siRNAs, then stimulated with nesfatin-1. IL-1β expression was examined by qPCR and ELISA. (**D**) Cells were incubated with nesfatin-1 for the indicated time intervals; p85 and Akt phosphorylation was examined by Western blot. (**E**–**F**) Quantitative data for p-p85 and p-Akt expression. ^*^p < 0.05 compared with the control group; ^#^*p* < 0.05 compared with the nesfatin-1-treated group.

OASFs are critical for maintaining homeostasis of the synovial microenvironment [29]. Treatment of OASFs with nesfatin-1 increased messenger RNA (mRNA) synthesis in a concentration-dependent manner (Figure 2A). Western blot and ELISA assays revealed that nesfatin-1 increased cellular and secreted IL-1β protein expression (Figure 2B–2D). The PI3K and Akt signaling cascade mediates IL-1β expression during the progression of arthritis [25]. Treating OASFs with either a PI3K inhibitor (Ly294002) or an Akt inhibitor (Akti) markedly antagonized nesfatin-1-induced IL-1β synthesis (Figure 3A and 3C). Similar results were observed when the cells were transfected with p85 or Akt small interfering RNAs (siRNAs) (Figure 3B and 3C). Nesfatin-1 treatment time-dependently promoted p85 and Akt phosphorylation in OASFs (Figure 3D–3F), indicating that the PI3K/Akt pathway regulates nesfatin-1-enhanced synthesis of IL-1β in human OASFs.

**Figure 4:**
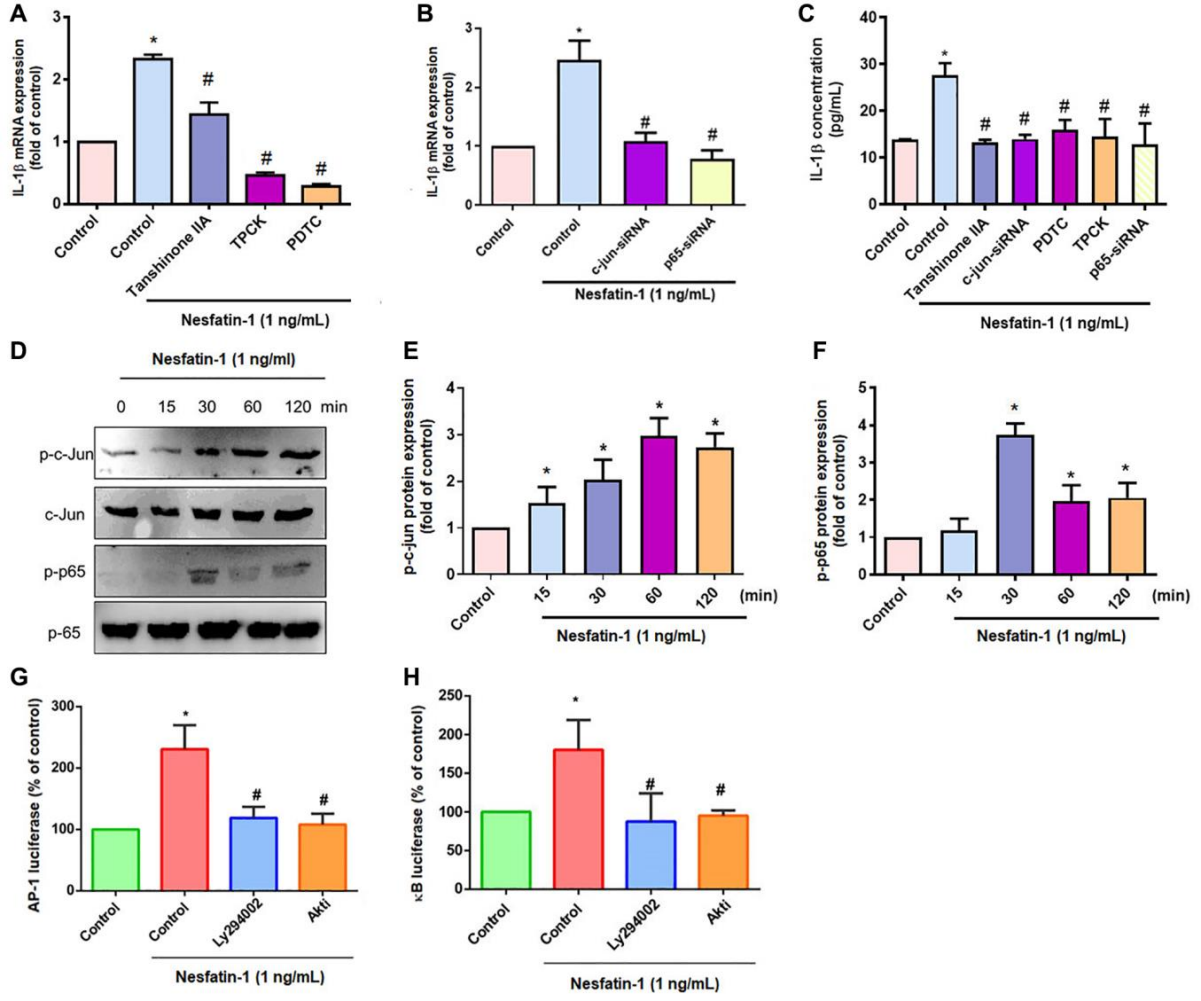
**The AP-1 and NF-κB pathways mediate nesfatin-1-induced stimulation of IL-1β.** (**A**–**C**) OASFs were treated with an AP-1 inhibitor (tanshinone IIA) or NF-κB inhibitor (PDTC and TPCK), or transfected with c-Jun or p65 siRNAs, then stimulated with nesfatin-1. IL-1β expression was examined by qPCR and ELISA. (**D**) Cells were incubated with nesfatin-1 for the indicated time intervals; c-Jun and p65 phosphorylation was examined by Western blot. (**E**–**F**) Quantitative data for p-c-jun and p-p65 were shown. (**G** and **H**) Cells were treated with indicated inhibitors then stimulated with nesfatin-1, and AP-1 and NF-κB luciferase activity was examined. ^*^p < 0.05 compared with the control group; ^#^*p* < 0.05 compared with the nesfatin-1-treated group.

AP-1 and NF-κB transcriptional activities regulate IL-1β-mediated inflammatory responses [33]. Transfection of OASFs with an AP-1 inhibitor (tanshinone IIA) or NF-κB inhibitors (PDTC and TPCK) effectively antagonized nesfatin-1-induced IL-1β expression (Figure 4A and 4C), as did siRNAs against c-Jun and p65 (Figure 4B and 4C), whereas nesfatin-1 facilitated the phosphorylation of c-Jun and p65 (Figure 4C–4F). Stimulation of OASFs with nesfatin-1 enhanced AP-1 and NF-κB luciferase activity, which was reversed by the PI3K and Akt inhibitors (Figure 4G and 4H). These results indicate that nesfatin-1 promotes AP-1 and NF-κB-dependent IL-1β production through the PI3K/Akt pathway.

### Inhibiting miR-204-5p expression regulates nesfatin-1-enhanced stimulation of IL-1β production in human OASFs

**Figure 5:**
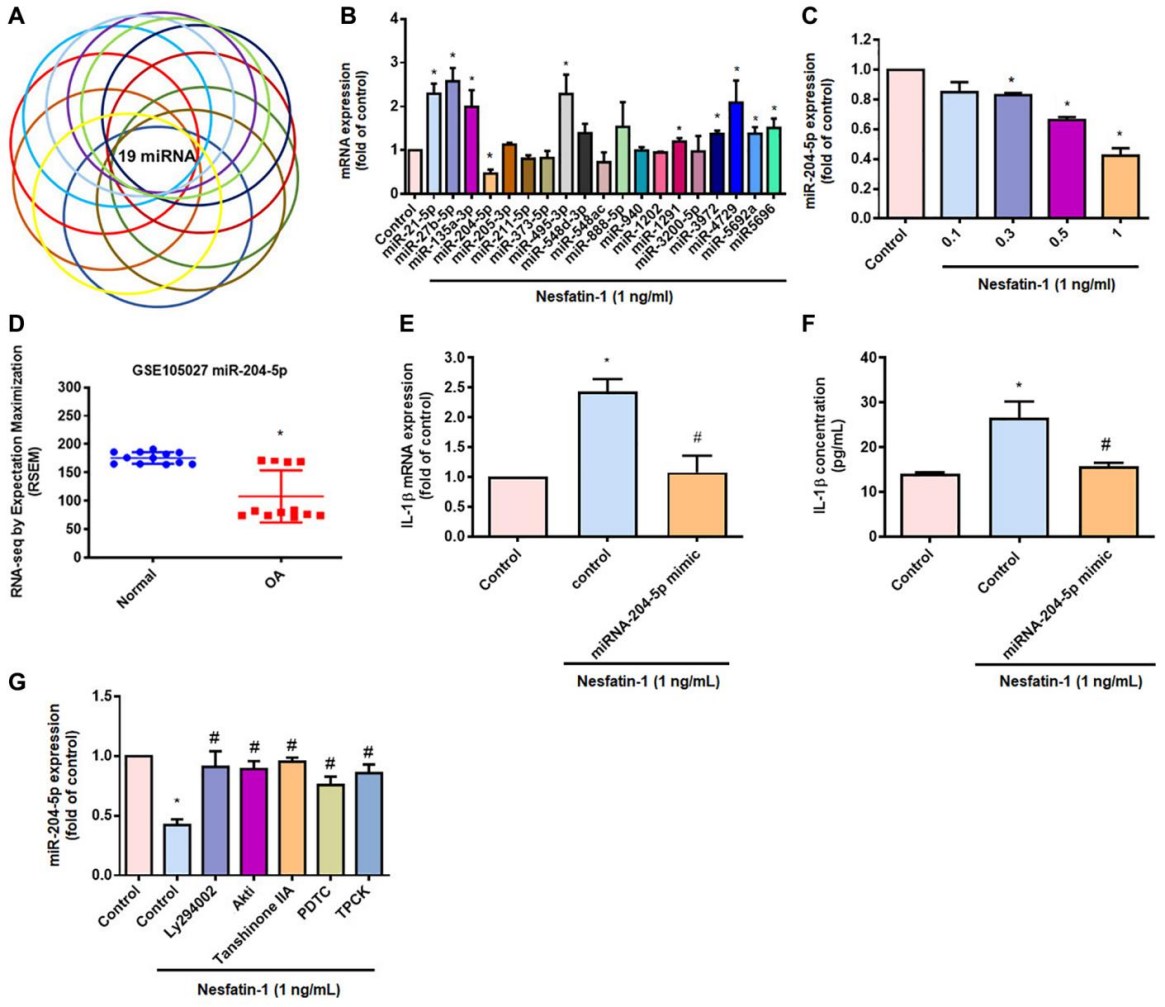
**Inhibiting miR-204-5p expression regulates nesfatin-1-induced stimulation of IL-1β production in human OASFs.** (**A** and **B**) miRNA target prediction software was used to identify miRNAs that potentially bind to the IL-1β 3’-UTR plasmid. (**C**) OASFs were incubated with nesfatin-1 and miR-204-5p levels were examined by qPCR. (**D**) Levels of miR-204-5p in normal and OA synovial tissues retrieved from the GEO dataset. (**E** and **F**) Cells were transfected with miR-204-5p mimic, then stimulated with nesfatin-1. IL-1β expression was examined by qPCR and ELISA. (**G**) Cells were treated with PI3K, Akt, AP-1 and NF-κB inhibitors, then stimulated with nesfatin-1 prior to qPCR analysis of miR-204-5p levels. ^*^p < 0.05 compared with the control group; ^#^*p* < 0.05 compared with the nesfatin-1-treated group.

The mediation of IL-1β synthesis by miRNAs is crucial in the progression of OA [25]. Our search of online databases for miRNA target prediction data suggested that the three prime untranslated region (3′-UTR) of IL-1β mRNA includes 19 promising candidate miRNAs (Figure 5A and 5B). Treatment of OASFs with nesfatin-1 significantly lowered miR-204-5p expression (Figure 5B) and, at the concentrations of 0.1, 0.3, 0.5 or 1 ng/mL, markedly inhibited miR-204-5p synthesis in a concentration-dependent manner (Figure 5C). The GEO dataset also revealed significantly lower levels of miR-204-5p in OA synovial tissue compared with tissue from healthy controls (Figure 5D). Transfection of OASFs with miR-204-5p mimic markedly inhibited nesfatin-1-promoted facilitation of IL-1β production (Figure 5E and 5F). Next, we observed that the PI3K, Akt, AP-1 and NF-κB inhibitors all antagonized nesfatin-1-induced inhibition of miR-204-5p synthesis (Figure 5G). These results indicate that nesfatin-1 increases IL-1β production by suppressing miR-204-5p synthesis in the PI3K, Akt, AP-1 and NF-κB signaling pathways.

## DISCUSSION

OA is well recognized for its characteristics of joint degradation and synovial membrane inflammation [34]. OASFs in the joint microenvironment are critical to the progression of OA, increasing proinflammatory cytokine production, which leads to cartilage degradation and bone erosion [34]. Numerous commercially available arthritis therapeutics target inflammatory cytokines, including IL-1β [35]. Adipokines are produced and secreted by adipose tissue and are involved in many physiological activities, including glucose and lipid metabolism, and also immune and inflammatory responses [36]. In OA, adipokines (including leptin, adiponectin and resistin) are considered to be useful therapeutic targets, as they can regulate proinflammatory cytokine expression and thus influence OA development [37–40]. A recent study reported that adiponectin is capable of inducing gene expression of monocyte chemoattractant protein-1 (MCP-1), IL-6 and matrix metalloproteinase 1 (MMP-1) in human OA chondrocytes [38], while leptin increases levels of IL-1, IL-8 and MMPs in chondrocytes as well as IL-6 and MMP-13 expression in synoviocytes, so contributes to cartilage catabolism in OA [41]. Resistin can activate the p38-MAPK, NF-κB and cyclic AMP (cAMP)-protein kinase A (PKA) signaling pathways and thus stimulate proinflammatory cytokine and chemokine expression in OA disease [42]. Nesfatin-1 has demonstrated anti-inflammatory activities [32], although the effect of nesfatin-1 on IL-1β production in OA is uncertain. Our study demonstrates that human OA synovial tissue contains higher levels of nesfatin-1 and IL-1β compared with tissue from healthy individuals. Cellular investigations revealed that nesfatin-1 promotes IL-1β synthesis. We also confirmed that nesfatin-1 increases IL-1β concentrations in human OASFs by suppressing IL-1β synthesis in the PI3K/Akt, AP-1 and NF-κB pathways.

PI3K/Akt activation is crucial in the adjustment of numerous cellular roles [43]. Notably, the PI3K/Akt signaling cascade mediates IL-1β expression during the progression of arthritis disease [25, 44, 45]. Our data show that nesfatin-1 promotes the phosphorylation of PI3K and Akt, while their respective inhibitors inhibit nesfatin-1-induced IL-1β production in human OASFs. This was confirmed by similar results with p85 and Akt siRNAs. Our evidence reveals that activation of PI3K/Akt signaling controls nesfatin-1-enhanced promotion of IL-1β synthesis during OA development.

Numerous transcription factor binding elements have been reported in the promoter site of IL-1β [46, 47]. In particular, AP-1 and NF-κB mediate IL-1β transcription and inflammatory responses [25, 46]. We found that inhibitors of AP-1 (tanshinone IIA) and NF-κB (PDTC and TPCK) suppressed nesfatin-1-induced production of IL-1β in human OASFs. Confirmation of these effects by genetic inhibition using c-Jun and p65 siRNAs indicated that AP-1 and NF-κB transcriptional activation is mediated by nesfatin-1-induced synthesis of IL-1β. We also observed that nesfatin-1 enhances c-Jun and p65 phosphorylation, as well as AP-1 and NF-κB luciferase activities. Pharmacological inhibitors of PI3K and Akt antagonized nesfatin-1-mediated activities, suggesting that nesfatin-1 promotes AP-1 and NF-κB-dependent IL-1β production and inflammatory responses through PI3K/Akt signaling.

miRNAs post-transcriptionally control gene synthesis [48]. During the development of arthritis disease, aberrant miRNA generation regulates inflammatory cytokine production and cartilage catalysis [48–50]. In particular, the mediation of IL-1β synthesis by miRNAs facilitates the progression of OA [25]. Our analysis of open-source databases identified 19 miRNAs that interfere with IL-1β transcription. Nesfatin-1 markedly inhibited levels of miR-204-5p expression. The analysis of records from the GEO database also found lower miR-204-5p levels in OA patients than in healthy controls. We enhanced miR-204-5p synthesis in human OASFs by transfecting them with a specific miR-204-5p mimic, which markedly inhibited IL-1β expression and cellular inflammatory responses. miR-204-5p expression was negatively associated with levels of IL-1β and proinflammatory cytokines in OA. Moreover, we found that inhibitors of PI3K (Ly2940002), Akt (Akti), AP-1 (tanshinone IIA) and NF-κB (PDTC and TPCK) can increase levels of miR-204-5p expression in human OASFs treated with nesfatin-1. Thus, the results indicated that miR-204-5p synthesis can be regulated by the PI3K/Akt pathway by facilitating the transcription of AP-1 and NF-κB into the nucleus of nesfatin-1-treated human OASFs. Thus, our evidence has identified that miR-204-5p exhibits novel anti-inflammatory properties, although the mechanisms of OA disease are complex and we only examined the *in vitro* effects of nesfatin-1 in human OASFs. Future work is needed to ascertain the *in vivo* effects of nesfatin-1 in OA. We suggest that further research should screen for a drug that can inhibit nesfatin-1 expression in inflammatory diseases such as OA.

**Figure 6:**
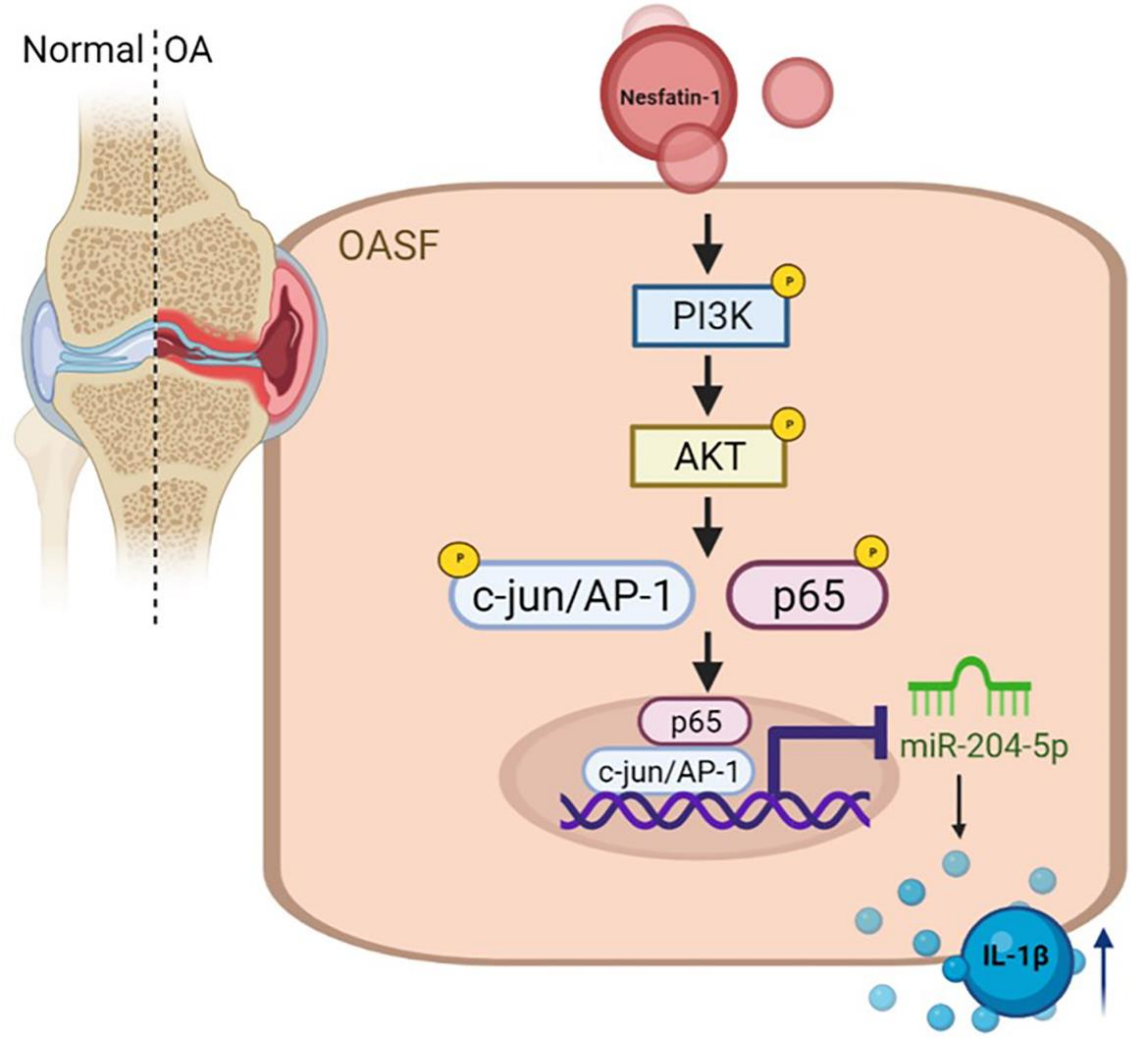
**Schema illustrating the effects of nesfatin-1 upon IL-1β synthesis during OA progression.** Nesfatin-1 promotes IL-1β synthesis in human OASFs by suppressing miR-204-5p synthesis in the PI3K, Akt, AP-1 and NF-κB signaling pathways.

In conclusion, our study has identified that nesfatin-1 facilitates IL-1β production in human OASFs by suppressing miR-204-5p synthesis in the PI3K/Akt, AP-1 and NF-κB pathways (Figure 6). We believe that targeting nesfatin-1 may assist with the management of OA disease.

## MATERIALS AND METHODS

### Materials

Nesfatin-1 (GTX00739, polyclonal), IL-1β (GTX74034, polyclonal), β-actin (GTX109639, polyclonal), p85 (GTX111068, polyclonal), Akt (GTX121937, polyclonal), c-Jun (GTX112974, polyclonal) and p65 (GTX102090, polyclonal) antibodies were purchased from GeneTex (Hsinchu, Taiwan). We bought ON-TARGETplus siRNAs from Dharmacon (Lafayette, CO, USA). qPCR primers and probes, as well as PCR Master Mix, were bought from Applied Biosystems (Foster City, CA, USA). The phosphorylated forms of p85 (17366, polyclonal), Akt (4060, polyclonal), c-Jun (9165, polyclonal) and p65 (3033, polyclonal) antibodies were bought from Cell Signaling (Danvers, MA, USA). AP-1 and NF-kB luciferase plasmids were purchased from Stratagene (La Jolla, CA, USA). TRIzol, a reverse transcription kit and Lipofectamine 2000 were purchased from Invitrogen (Carlsbad, CA, USA). Recombinant human nesfatin-1 and the IL-1β ELISA kit (900-K95) were purchased from PeproTech (Rehovot, Israel). Other chemicals not already mentioned were bought from Sigma-Aldrich (St. Louis, MO, USA). Pharmacological inhibitors for PI3K (1 μM LY294002 catalog number: 440202), Akt (1 μM Akti, catalog number: A6730), c-Jun (10 μM, Tanshinone IIA catalog number: T4952), p65 (3 μM TPCK catalog number: T4376; 1 μM PDTC, catalog number: P8765) were supplied by Sigma-Aldrich (St. Louis, MO, USA).

### Cell culture

OASFs were obtained from OA patients synovial tissues using collagenase (10 %) treatment and cultured in Dulbecco's Modified Eagle Medium (DMEM; Invitrogen) contain 10% (v/v) fetal bovine serum (FBS), 50 U/L penicillin, 50 μg/mL streptomycin and glutamine, and were maintained at 37°C in a humidified atmosphere of 5% CO_2_, as described in previous studies [44].

### Bioinformatics analysis

Gene level profiles in OA and normal synovial tissue were obtained from the GEO database and analyzed for levels of nesfatin-1 and IL-1β expression.

### Collection of clinical samples

Study approval by the Institutional Review Board (IRB) of China Medical University Hospital and all participants provided written informed consent before study enrolment (Approval Number: MUH108-REC3-039). Clinical samples were taken from 8 patients undergoing total knee arthroplasty for OA and from 8 patients undergoing arthroscopy after trauma/joint derangement (who served as healthy controls).

### Transfection siRNA and miRNA mimic

OASFs were transfected with siRNAs (control, PI3K, Akt, c-Jun and p65) or miR-204-5p mimic (20 μM) for 24 h using Lipofectamine 2000 (Invitrogen, Waltham, MA USA), as described in our previous study [51].

### Western blotting analysis

SDS-PAGE resolved the total proteins, which were then transferred to the PVDF membranes, as per our previous publications [52–55]. The PVDF membranes were blocked with 4% non-fat milk in PBST for 1 h before incubating them with primary antibodies for 1 h, followed lastly by 1 h of incubation with HRP-conjugated secondary antibodies. Finally, we examined the immunoblot band using an imaging system (ImageQuant™ LAS 4000).

### q-PCR analysis

Total RNA was extracted from OASFs with TRIzol agent and then transformed into complementary DNA using a reverse transcription kit. We conducted the qPCR analysis using PCR Master Mix. We used the SYBR^®^ RT-PCR kit and Mir-X™ miRNA First-Strand Synthesis for reverse transcription of miRNAs. Analysis followed previous protocols [56–58].

### ELISA assay

OASFs were treated with pharmacological inhibitors or transfected with siRNAs before being treated with nesfatin-1 for 24 h. IL-1β levels in the medium were then examined using the IL-1β ELISA kit, according to the manufacturer’s procedures.

### Luciferase assay

OASFs were transfected with AP-1 or NF-κB luciferase plasmids (Stratagene; St. Louis, MO, USA) using Lipofectamine 2000, then stimulated with pharmaceutical inhibitors and nesfatin-1. Luciferase activity was examined according to our previous reports [5, 59, 60].

### Statistical analysis

Values are shown as the mean ± standard deviation (S.D.). Significant differences between each group were assessed by the Student’s *t*-test. All *p* values <0.05 were considered to be significant.
